# Calculation of the Geometries and Infrared Spectra of the Stacked Cofactor Flavin Adenine Dinucleotide (FAD) as the Prerequisite for Studies of Light-Triggered Proton and Electron Transfer

**DOI:** 10.3390/biom10040573

**Published:** 2020-04-09

**Authors:** Martina Kieninger, Oscar N. Ventura, Tilman Kottke

**Affiliations:** 1 CCBG, DETEMA, Facultad de Química, Isidoro de María 1616, 11800 Montevideo, Uruguay; oscar.n.ventura@gmail.com; 2 Department of Chemistry, Physical and Biophysical Chemistry, Bielefeld University, Universitätsstr. 25, 33615 Bielefeld, Germany; tilman.kottke@uni-bielefeld.de

**Keywords:** stacked flavin adenine dinucleotide (FAD) in microsolvation, vibrational spectra, supramolecular orbitals

## Abstract

Flavin cofactors, like flavin adenine dinucleotide (FAD), are important electron shuttles in living systems. They catalyze a wide range of one- or two-electron redox reactions. Experimental investigations include UV-vis as well as infrared spectroscopy. FAD in aqueous solution exhibits a significantly shorter excited state lifetime than its analog, the flavin mononucleotide. This finding is explained by the presence of a “stacked” FAD conformation, in which isoalloxazine and adenine moieties form a π-complex. Stacking of the isoalloxazine and adenine rings should have an influence on the frequency of the vibrational modes. Density functional theory (DFT) studies of the closed form of FAD in microsolvation (explicit water) were used to reproduce the experimental infrared spectra, substantiating the prevalence of the stacked geometry of FAD in aqueous surroundings. It could be shown that the existence of the closed structure in FAD can be narrowed down to the presence of only a single water molecule between the third hydroxyl group (of the ribityl chain) and the N7 in the adenine ring of FAD.

## 1. Introduction

Flavin cofactors, like flavin adenine dinucleotide (FAD), are important electron shuttles in living systems. As components of flavoenzymes, flavin cofactors catalyze a wide range of one- or two-electron redox reactions. In addition, flavoproteins also act as photoreceptors and photoenzymes. Photoreception involves mostly three families of flavoproteins: LOV (light, oxygen, voltage) proteins [1], cryptochromes [2] and BLUF (sensors of blue light using flavin) proteins [3]. These proteins act as blue-light-receptors in many organisms. Moreover, cryptochrome has been found to enable flies to detect magnetic fields [4] and has been discussed in connection with a putative role in the magnetoreception of migratory birds [5]. Photoenzymes with flavin cofactor are DNA photolyases, which repair UV light-damages of DNA [6], and the fatty acid decarboxylase [7]. In many flavoproteins, the cofactor is not covalently bound but in a dynamic equilibrium with the solution [8].

For FAD, different conformations have been found. In aqueous solution, predominantly a “stacked”, closed conformation of isoalloxazine and adenine rings of FAD is present and attributed to π–π interactions [9]. In most enzymes, BLUF and some LOV proteins, the FAD is bound in the extended, open conformation. In cryptochromes and photolyases, FAD adopts a “U-shaped” conformation in between the open and closed conformation.

To gain deeper insight in the mechanisms of the electron transfer within flavoproteins and in solution, the study of FAD—experimental and computational—is an ongoing essential prerequisite. Experimental investigations done with respect to flavin cofactors include UV/vis, fluorescence and infrared spectroscopy. The excited-state behavior of FAD has been studied in aqueous solution (D_2_O) by employing time-resolved fluorescence up-conversion and transient absorption spectroscopy [10,11]. They found that FAD in aqueous solution exhibits significantly shorter excited state lifetime than its analog flavin mononucleotide (FMN). This result was explained by the presence of the “stacked” FAD conformation. The reason for the fast deactivation of the excited state in FAD has been explained by the existence of an intramolecular electron transfer from adenine to the isoalloxazine.

In the infrared spectral range, the cofactors FAD and flavin mononucleotide (FMN) were investigated in aqueous medium (H_2_O) by Fourier transform infrared spectroscopy [12]. Transmission and attenuated total reflection (ATR) configuration were employed in direct comparison. Absorption spectra in the range of 920–1800 cm^−1^ were determined and the carbonyl vibrations were resolved at 1661 and 1712 cm^−1^. As stated by Spexard et al. for FAD [12], the vibrational spectrum of flavin overlaps with the spectrum of adenine. Additional stacking of the isoalloxazine and adenine rings should occur, which might be visible as an influence on the vibrational modes [12]. This assumption is substantiated by the observations of Li et al. [13] using time-resolved mid-IR transient absorption spectroscopy. This study provided evidence for an electron transfer from adenine to isoalloxazine by a bleach of the adenine stretch at 1623 cm^−1^, which rises with 1.1 ps and decays with 9 ps. The addition of polar aprotic solvents such as dimethyl sulfoxide (DMSO) is altering the IR-spectra detected to resemble those of FMN, which was interpreted as a breaking of the π-stacked complex and production of a predominantly “open” conformer with a long excited-state lifetime. The model of Li et al. [13,14] has been adopted by Sengupta et al. [15] in their investigation of pH-dependent dynamic behavior of FMN and FAD on a femtosecond to nanosecond time scale. More recently the pH-depending prevalence of the stacked form of FAD proposed by Li et al. [13] has been used as a model concept in the publication of Bubniene et al. [16] about the fluorescence quenching-based evaluation of a glucose oxidase composite with a conducting polymer—polypyrrole: The stacked form of FAD is thought to be responsible for the fast quenching of the FAD fluorescence, which could not unfold freely in this specific environment as the authors stated. 

Despite theoretical studies which include lumiflavin, riboflavin and FMN as a model of FAD–flavoprotein interactions in proteins [17,18,19,20], theoretical studies of the stacked form of FAD as well as studies of the unfolding process from the closed to the open conformer are still missing. The present work aims to fill this gap.

## 2. Materials and Methods

Molecular dynamic (MD) [21] runs were performed with both, open and closed structures using AMBER [22] in water (1000 water molecules surrounding the FAD). The conditions employed were 100 ps with 500 ps runtime at a step size of 0.01 ps, the starting temperature was 0 K, the simulation temperature 298 K. In order to compare the structures of FAD in aqueous solution with structures which may be adopted by FAD in polar aprotic solvents, the MD calculations were repeated with DMSO (replacing the water molecules by DMSO molecules). The resulting structures of the MM calculations were taken as starting points of geometry optimizations with the semi-empirical PM6 method—starting with 100 explicit solvent molecules (water and DMSO, respectively). This initial step was followed by calculations which gradually reduced the number of the solvent molecules in order to build models of FAD in solution and microsolvation while keeping the geometrical properties of the solvent-FAD system intact. The resulting systems served as starting geometries of FAD in microsolvation for density functional theory (DFT) calculations [23].

DFT studies on FAD-solvent systems, e.g., in microsolvation with up to 15 explicit water molecules and using the continuous surface charge PCM method followed. The M06 potential [24] with the 6-31G (d,p) basis set and the ultrafine grid was used to perform vibrational studies of the FAD-water systems.

## 3. Results

### 3.1. MD Calculations to Obtain Starting Geometries of FAD in the Closed Geometry

**Figure 1:**
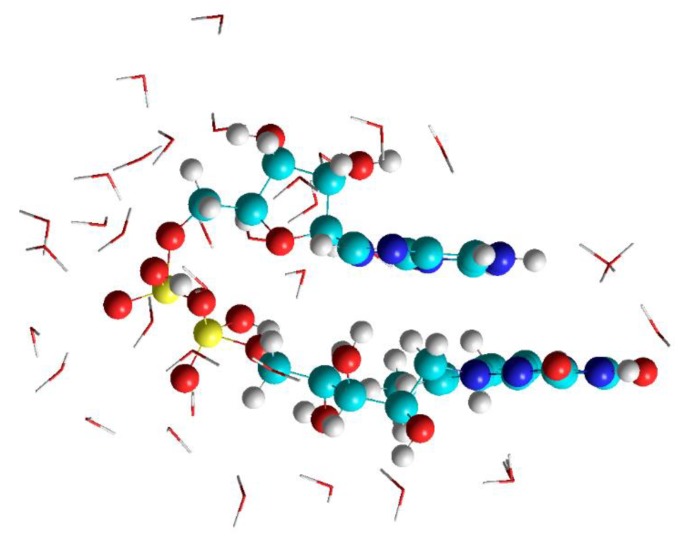
Result of the molecular dynamic (MD) calculation of flavin adenine dinucleotide (FAD) in water using AMBER.

**Figure 2:**
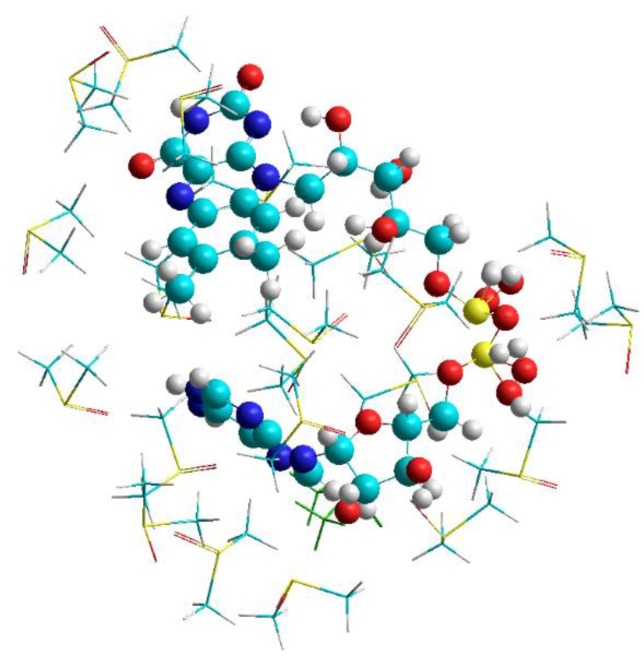
Result of the MD calculation of FAD in dimethyl sulfoxide (DMSO) using AMBER.

The calculations were performed in order to provide starting geometries for the calculations of FAD-water complexes with DFT methods. The focus was not on observing the course of geometry changes and folding in FAD during MD. Instead the focus was on FAD in water (H_2_O) to provide starting geometries for reproducing the IR spectrum. As a starting geometry of the open conformation, the geometry of FAD as a free ligand was chosen as it is realized in 1948 systems and listed in the PDB database (https://www4.rcsb.org/ligand/FAD). The closed structure was obtained by scanning the dihedral angles in the ribityl and adenosine-phosphate part of the open FAD in search of a structure with parallel planes of adenine and isoalloxazine. The open as well as the closed FAD were surrounded by 1000 water molecules using the features of the periodic boundary conditions inside the Hyperchem Program. Both structures, the open as well as the closed FAD in water maintained their structures throughout the runtime (500 ps) of the MD calculations (Figure 1 shows the situation for the closed FAD). Replacing water by the polar aprotic solvent DMSO (i.e., replacing the water molecules by DMSO molecules) did not change the geometry of the open FAD, but immediately opened the closed structure (Figure 2).

This first step was inspired by Visser et al. [25], who detected basically 4 different closed structures of FAD. Since each two of them are basically identical and since the “upside down” geometry (with respect to the geometry shown in Figure 1) stacks the hydrophobic part of the isoalloxazine with the amine-group of adenine, the “upside down geometry” opens during the optimization on the DFT level of theory and was therefore discarded from further investigations.

In a second step water molecules in a more than 2.5 Å distance from the FAD were removed, the resulting geometries (open and closed FAD) were optimized on the semiempirical level (PM6). To obtain starting geometries for the subsequent modelling of FAD in micro solvation and the calculation of the respective IR spectra with DFT, the number of participating water molecules was even further reduced: In the end the model included water molecules at the positions N1, C2, N3, C4, and N5 of the isoalloxazine moiety, one water molecule between N7 of adenine and the ribityl chain which turned out to be very important for keeping the FAD structure in its closed geometry, two additional water molecules as a second water clip between the amine group of adenine and the isoalloxazine ring at C4 as well as a third water clip between the amine of adenine and C2 and N1, respectively.

In the following the geometry and the role of each of the water molecules in the FAD-water complex will be shown and discussed.

### 3.2. DFT Studies on the Closed Complex FAD-Water and the Role of the Water Molecules in the FAD-Water Complex

#### 3.2.1. The First “Water Clip”

**Figure 3:**
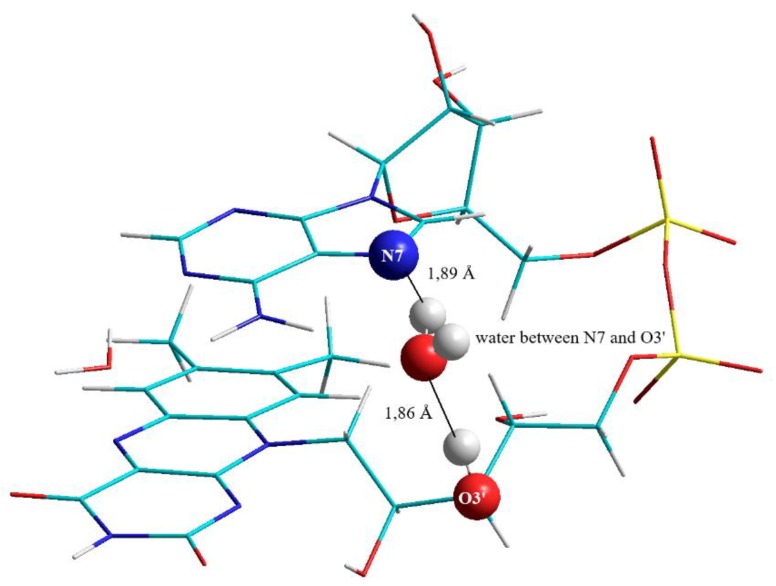
Water clip 1 is formed by a water molecule between N7 of adenine and the O3′ hydroxyl of the ribityl chain.

The water molecule between the adenine and the isoalloxazine forms the clip which is responsible for keeping the FAD in the correctly folded structure (shown in Figure 3): This water molecule is located between the third hydroxyl group (of the ribityl chain) and the N7 in the purine ring: The distance between the oxygen of the “clipping” water and the hydrogen of the hydroxyl group at position O3′ in the ribityl chain of the riboflavin moiety amounts to 1.86 Å, the distance between the hydrogen of the “clipping” water and N7 of purine in the ADP moiety is 1.89 Å, respectively.

Without the presence of this water molecule FAD will adopt an open structure: This was tested by removing the water molecule located between N7 and the ribityl chain from the FAD-water complex. Performing a new geometry optimization of the otherwise unchanged FAD-water complex shows that the FAD will adopt a T-shaped open conformation: The vibrational spectrum of this open structure was compared with the closed structure (see “Vibrational spectra”).

#### 3.2.2. The Second “Water Clip”

A second “clip” in the FAD-water complex was detected, which is less important for keeping the FAD-water complex in a closed structure. This was verified by removing the second clip while the “first water-clip” between the adenine N7 and the ribityl was kept intact: The structure was keeping its closed geometry without the second “water clip”.

**Figure 4:**
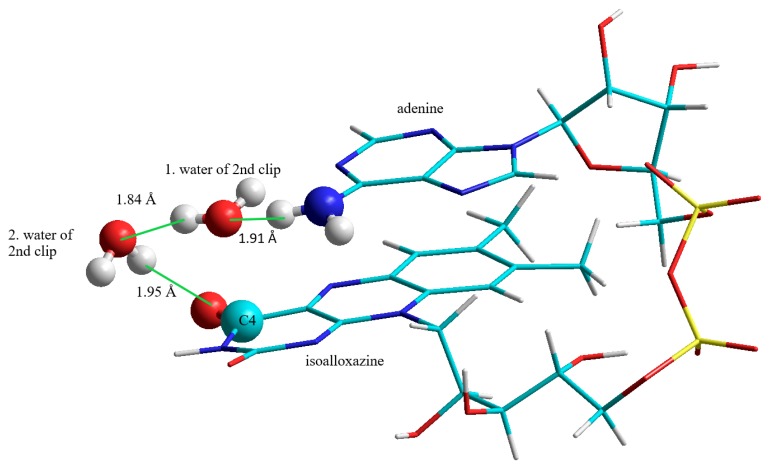
FAD-water complex formed by two water molecules bridging the C4O of isoalloxazine and the amino group (N6A) of adenine.

This second “water-clip” consists of two water molecules and connects the adenine to the isoalloxazine in the following way: The first water molecule is bound to the amino-group of the adenine, the second water molecule spans the bridge between the water-adenine-complex and the C4O group of the isoalloxazine (Figure 4):

#### 3.2.3. Water Molecules Surrounding Isoalloxazine at the C2, N3, C4, and N5 Positions

**Figure 5:**
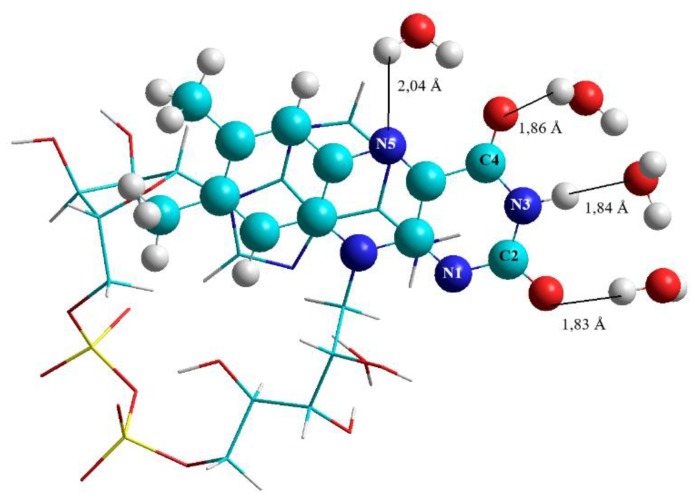
Microsolvation with explicit water at positions C2O, N3H, C4O, and N5 of the isoalloxazine ring.

As shown in Figure 5, this model was completed by adding water molecules surrounding the isoalloxazine at the positions C2O, N3H, C4O, and N5. The additional water molecules at C2O and C4O are important for calculating the vibrational spectrum since they may shift the bands compared to a spectrum calculated without solvation at these positions.

#### 3.2.4. The Third “Water Clip”

**Figure 6:**
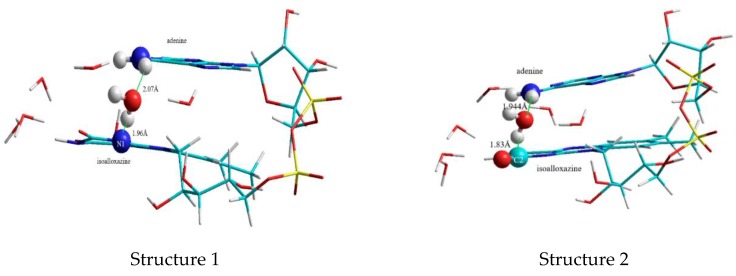
Structures of stacked FAD with clip 3, a water molecule bridging the N6A amine group of adenine and N1 (structure 1) or C2=O of isoalloxazine (structure 2).

To model a FAD complex able to perform intramolecular electron transfer suggested by Li and Glusac [13,14] the FAD-water model shown above in Figure 4 was enhanced by including an additional water molecule between the amine-group of adenine and N1 in isoalloxazine of the complex (Figure 6). This additional third water clip proved to be important since it lowers the distance between the planes substantially from 3.81 Å (for the model without this specific water molecule) to 3.3 Å and 3.15 Å for the two structures resulting:

In structure 1 the water molecule connects the amine group of adenine with the N1 of isoalloxazine, while the water in structure 2 links the amine group with C2O.

**Figure 7:**
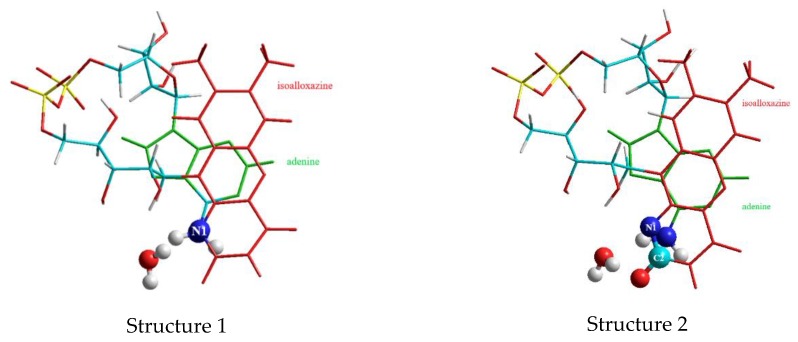
Overlap of the pyrimidine ring of adenine (green) with the pyrazine ring (ring II) of isoalloxazine (red).

On both structures geometry optimizations have been performed in the singlet as well as in the triplet state: The total energies (in atomic units) of the triplet states are about 50 to 30 kcal higher than the corresponding singlet states (Table 1):

The formation of the hydrogen bond between C2O and the amine group of adenine instead of forming the hydrogen bond with N1 means a shifting of the two heterocycles of adenine and isoalloxazine in FAD towards a higher overlap of the two planes as shown in Figure 7.

**Figure 8:**
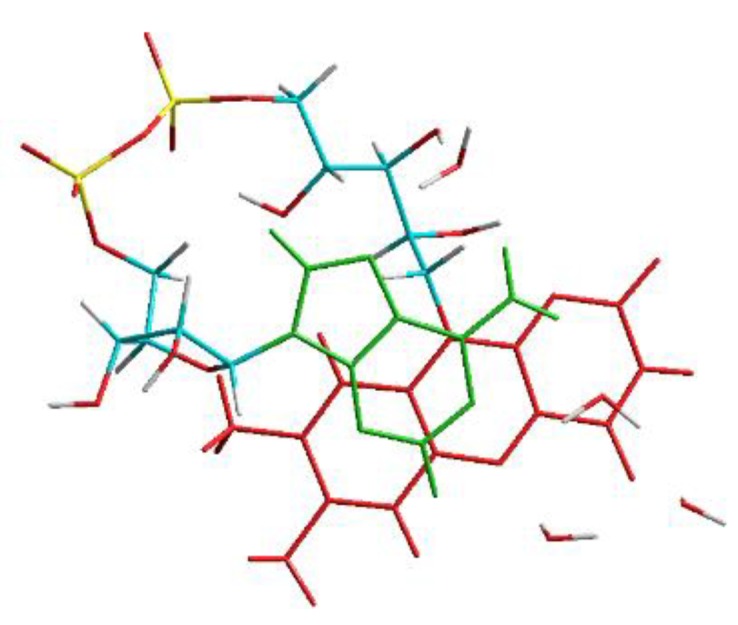
Overlap of the ring systems without the 3rd water clip: adenine (green) and isoalloxazine (red).

This is reflected by the distance between the isoalloxazine and the adenine which amounts to 3.3 Å for structure 1 and 3.15 Å for structure 2. Nevertheless, the third water clip in both structures improves the overlap of the two ring systems substantially (Figure 8):

More important than the interplanar distances are the changes in the distances between C4a and C10a, N1, and C2O as well as between C4O and N5 in the isoalloxazine ring. Those distances depend only on the state (triplet or singlet) and were found to be independent of the geometry (structure 1 and structure 2). The distances are compared with the results for the FAD-water complex without the clipping water from the third “clip”. The values are given in the following Table 2:

#### 3.2.5. Molecular Orbitals—HOMO/LUMO

**Figure 9:**
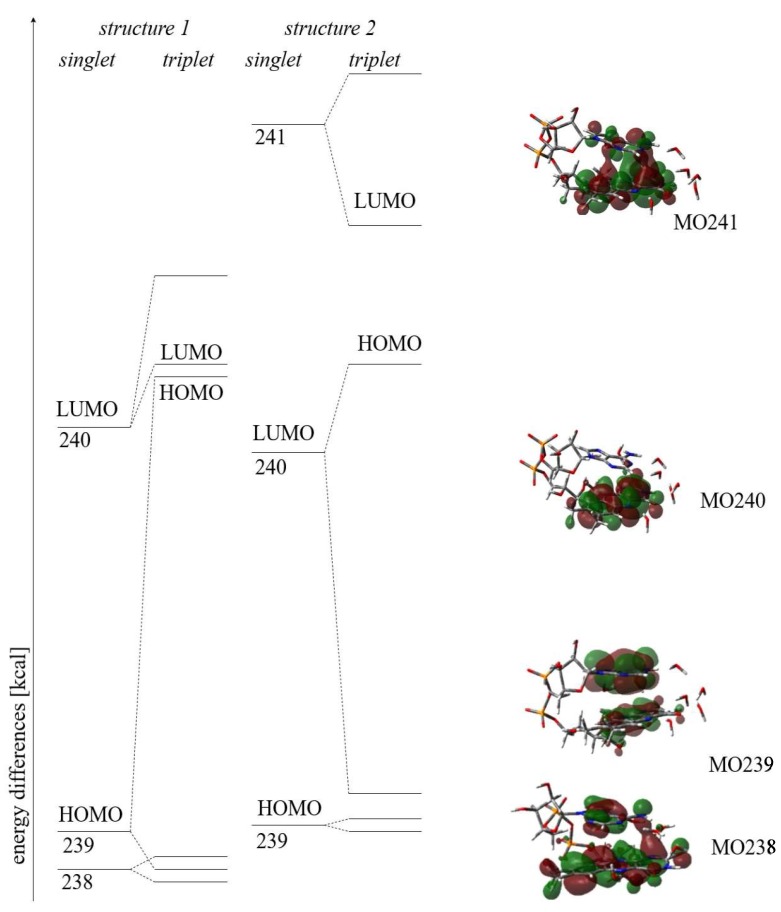
MOs 238–241 of the singlet/triplet in structure 1 and 2.

Since the geometrical properties of the FAD-water complex may be reflected by the molecular orbitals, the LUMO and HOMO as well as other molecular orbitals of interest are shown in the following graph according to their relative energies (Figure 9): Structure 1 refers to the geometry including the hydrogen bridge between the amino group of adenine towards the N1 in isoalloxazine, while structure 2 refers to the geometry featuring the hydrogen bridge between the amino group of adenine towards the C2O in isoalloxazine.

The corresponding energies (in atomic units a.u.) are shown in the following Table 3.

#### 3.2.6. Supramolecular Orbitals

**Figure 10:**
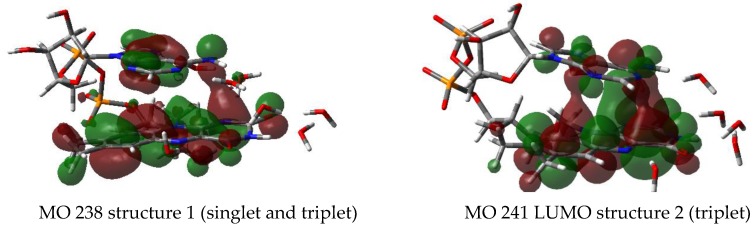
Supramolecular orbitals in closed FAD-water complexes.

Only FAD-water complexes with the 3rd water clip between the amine group of adenine and N1 or C2O show the two supramolecular orbitals found in those structures (Figure 10). While MO 241 has been identified as the LUMO in one of the triplet states, the supramolecular MO 238 plays not such a role as HOMO. Nevertheless, MO 238 encompasses MO 239 in the triplet of structure 1, the energy of −0.2399 a.u. for MO 239 compares with −0.2383 a.u. for MO 238 (or 0.8 kcal).

**Figure 11:**
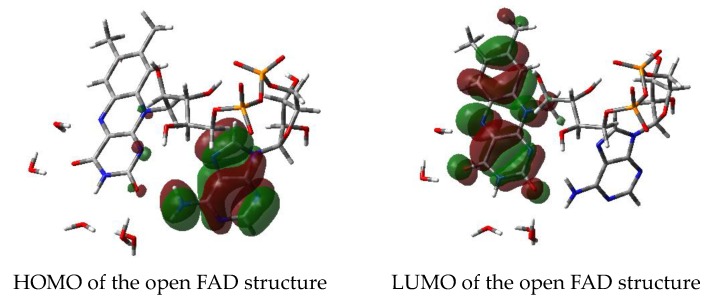
HOMO/LUMO of open FAD-water complexes.

Neither the open FAD nor the closed structure without the third water clip show this behavior: Instead the HOMO is confined to the adenine moiety while the LUMO occupies solely the isoalloxazine ring system (Figure 11).

#### 3.2.7. Vibrational Spectra

**Figure 12:**
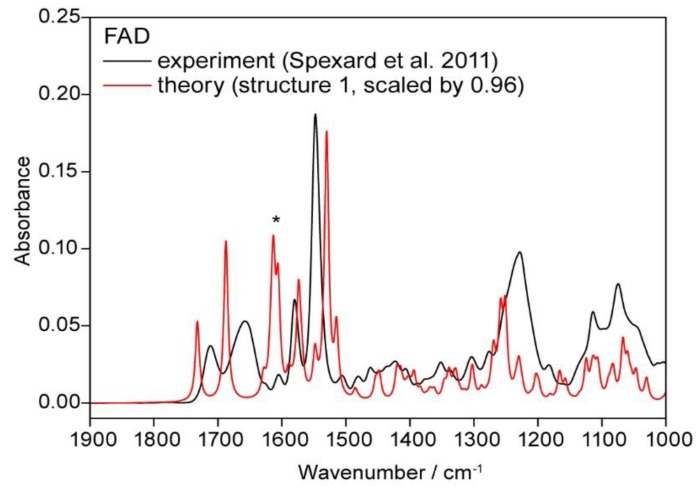
Comparison of the calculated spectrum of FAD with the experimental spectrum recorded in H_2_O. The peak signed with * contains contributions from water clip 1.

The comparison of the experimental IR-spectrum with the calculated vibrational spectrum is given in the following graph (Figure 12):

The theoretical values show overall good agreement with the experiment. The peak in both spectra (experimental and theoretical) at 1600 (signed with *) has to be attributed to vibrations caused by water clip 1 (between N7 of adenine and the ribityl chain) and the adenine (amine group and the imidazole moiety): The calculation overestimates the contribution of this vibration due to some bulk effect of the surrounding water molecules. 

**Figure 13:**
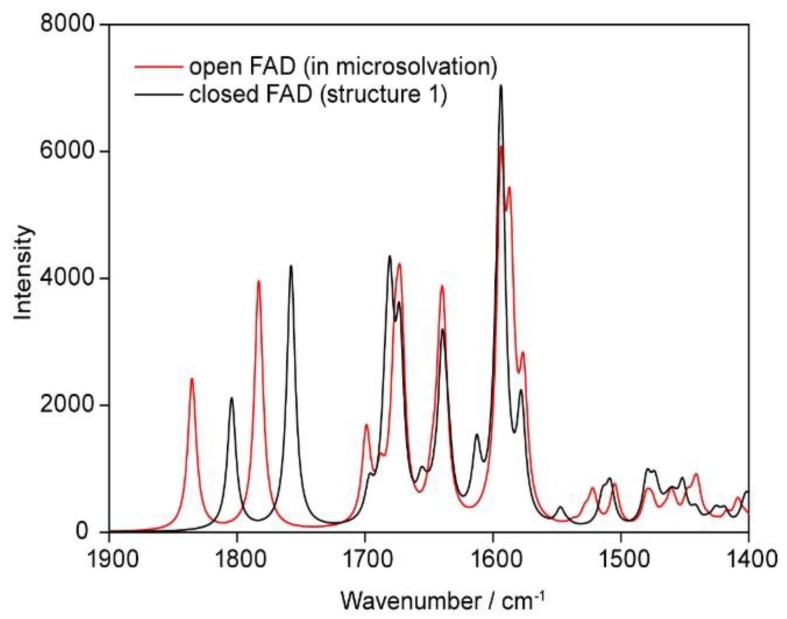
Calculated spectra of FAD in the open and closed forms.

**Figure 14:**
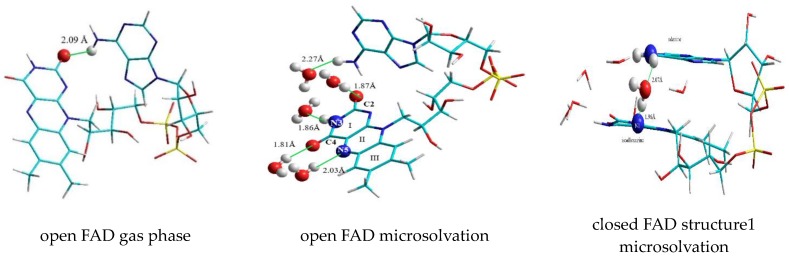
Geometries corresponding to the structures of Table 4.

The vibrational spectrum of the open FAD has been calculated in order to compare it with the spectrum of the closed form, the comparison between both forms are shown in the following graph (Figure 13):

The values of the most prominent vibrations (closed versus open FAD) are listed in the Table 4, the values have been scaled by a factor of 0.96. The open geometry was obtained by removing all water clips of the closed form: Optimizing this geometry resulted in an open, T-shaped conformation. In order to compare this open structure with the closed FAD-water complex in microsolvation, water molecules have been added to the “gas phase” open FAD at the positions C2, N3, C4, and N5 of the isoalloxazine and the amine group of adenine, geometry optimization was performed on this structure. The corresponding geometries of the open and closed FAD in gas phase and in microsolvation are shown in Figure 14.

**Figure 15:**
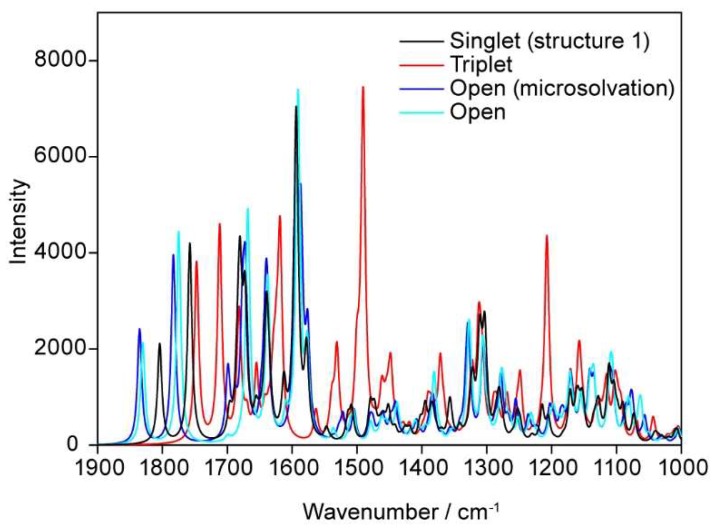
Comparison of calculated spectra of FAD including the triplet state.

The following graph provides a compilation of all the spectra which have been calculated, e.g., the open structures with and without solvating water molecules as well as the closed FAD-water complex in its singlet and triplet state (Figure 15). The most prominent band for the triplet at 1490 cm^−1^ has to be attributed to concerted vibrations of the isoalloxazine system together with vibrations in the adenine moiety: This type of coupled movement has not been detected when calculating the spectra related to all the other geometries of FAD or FAD-water complexes.

## 4. Discussion

The main purpose of this study was the determination of the geometry of FAD in aqueous environment to explain the experimental spectroscopical data of Spexard et al. [12]. Our model of the closed FAD structure in microsolvation is able to reproduce the prominent bands of the infrared spectrum, the C2=O and the C4=O stretches as well as the bands due to the stretches in the isoalloxazine moiety. The vibrational spectrum of the triplet shows the typical shift with respect to the singlet, e.g., 1490 cm^−1^ compared to 1594 cm^−1^. While the singlet includes only the vibrations in the isoalloxazine, the peak at 1490 cm^−1^ (triplet) shows an involvement of both moieties, the adenine and the isoalloxazine.

The majority of computational studies on FAD in the past have been dedicated to flavoproteins with FAD adopting T-shaped and other “open“ geometries, the “stacked“ geometry of FAD has not yet been the subject of those studies. The recent publication of Bubniene et al. [16] reflects this gap. According to Bubniene et al. differences in the FAD and polypyrrole average fluorescence relaxation time showed that the FAD composite with polypyrrole effectively quenched the FAD fluorescence since FAD could not freely unfold in this environment. Moreover they state that the intramolecular electron transfer took place between adenine and isoalloxazine moieties over the first 5 ps after the excitation. However, the FAD models of Bubniene et al. remain schematic and rely on the qualitative reasonings of Li et al. [13]. The same holds true for the study of Kao et al. [10]: Their “stacked” FAD geometry resembles more to an open or T-shaped geometry than to a closed structure.

Although being interested mainly in reproducing the experimental data of Spexard et al. [12], our attention was turned to the possibilities for obtaining the π-stacked complex suggested by Li et al. in 2008 [13]. In general the π-stacked complex shows the following properties: It depends indeed on the presence of the aqueous environment, featuring three important hydrogen-bonds. The most important “water clip” consists of the water between N7 of adenine and the ribityl moiety which fixes the position of the isoalloxazine with respect to the ADP backbone and keeping the two aromatic ring systems parallel in an adequate distance for a possible charge transfer. The second “water clip” consists of two water molecules and spans the distance between the amine group of adenine and the C4O of the isoalloxazine. These two water molecules are part of the whole microsolvation “water network” around the isoalloxazine moiety at C2O, N3 and C4O and are one of the reasons for the overall good agreement of the calculated vibrational bands with the experimental data. The third “water clip” may play a role in the fast quenching of FAD. As Wu et al. [26] stated in their studies about mechanisms of photoreactivity in hydrogen-bonded adenine–H_2_O complexes, the amine group of adenine acts as an H donor.

Our studies involved the singlet and triplet state of FAD: While the singlet state remains in the geometry of the oxidized FAD, the results for the triplet state show a shortening of the C4a-C10a bond while elongating the C4a-N5 and C10a-N1 bonds in the FAD-water complex, these properties tend to the geometry of the reduced FAD.

The most interesting result of studying the closed FAD-water complex consists in finding the supramolecular orbitals 238 and 241 in the triplet: Energetically MO 238 overlaps with MO 239 (HOMO), while the charge transfer involves MO 241 (LUMO). As stated before, the supramolecular orbitals originate from the closed FAD-water complexes specifically including the water molecule between adenine and isoalloxazine (e.g., water clip 3)—without the third water clip the supramolecular orbitals are not found. 

Moreover, the triplet shows reduced HOMO-LUMO gaps with respect to the singlet: While the energy difference between the orbitals 239 (HOMO) and 240 (LUMO) in the singlet amounts to 81 kcal in structure 1 and 73 kcal in structure 2, respectively, the gap between HOMO (239) and LUMO (240) is reduced to 4 kcal for the triplet in structure 1. In both cases (in the triplet as well as in the singlet) the HOMO (MO 239) involved is extended over both planes of the FAD, namely the isoalloxazine and the adenine. Most interestingly the triplet in structure 2 shows an involvement of the supramolecular orbital 241 which becomes the new LUMO with an energy difference to MO 240 (the “new” HOMO) of 24 kcal. Since the supramolecular orbital 241 (Figure 10) extends over the isoalloxazine and the adenine, while MO 240 remains located in the isoalloxazine moiety, the process of the charge transfer towards the isoalloxazine seems to be turned upside down by describing MO 240 as the “new” HOMO: This “reordering” of the molecular orbitals involved in the charge transfer may be due to the relatively modest basis set combined with the exchange correlation functional chosen and should be checked including larger basis sets and the global hybrid functional with 54% HF exchange.

## 5. Conclusions

Our calculations of the vibrational spectra of FAD water complexes show that the vibrational bands in the IR spectrum can be attributed to the closed and “π-stacked” structure of the FAD-water complex. The existence of the closed structure depends on the water between N7 of adenine and the ribityl backbone of FAD which fixes the planes of the adenine and the isoalloxazine in the adequate distance for a possible charge transfer.

## Figures and Tables

**Table 1:** Total energies of structure 1 and structure 2 in the singlet and triplet states in a.u. Energy differences between the structures as well as the multiplicities are given in kcal.

	Singlet	Triplet	Diff (Triplet−Singlet)
Structure 1	−3885.38051400 a.u.	−3885.30052427 a.u.	50.19 kcal
Structure 2	−3885.37366640 a.u.	−3885.31405627 a.u.	37.41 kcal
Diff (Struc1−Struc2)	−4.29 kcal	−8.49 kcal	

**Table 2:** Shortening and elongation of bonds in the triplet vs. singlet state.

flavin ring: N1, C10a, C4a, N5 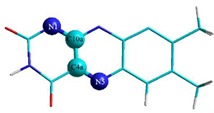	flavin C4a – C10a 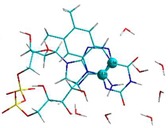	flavin C4a – N5 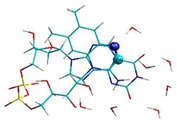	flavin C10a-N1 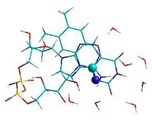
FAD triplet	1.40 Å	1.35 Å	1.32 Å
FAD singlet	1.44 Å	1.30 Å	1.30 Å
FAD without water clip	1.44 Å	1.31 Å	1.29 Å

**Table 3:** Energies of MOs 238–241 of the singlet/triplet in structure 1 and 2.

	Structure 1		Structure 2	
MO	Singlet [a.u.]	Triplet [a.u.]	Singlet [a.u.]	Triplet [a.u.]
238	−0.2473	−0.2511 −0.2383		
239	−0.2360 **HOMO**	−0.2399 −0.1785 **HOMO**	−0.2356 **HOMO**	−0.2398 −0.1785
240	−0.1059 **LUMO**	−0.1722 **LUMO** −0.0854	−0.1188 **LUMO**	−0.1722 −0.0852 **HOMO**
241			−0.0417	−0.0454 **LUMO** −0.0329

**Table 4:** Selected calculated frequencies of vibrations of FAD.

Open FAD (gas phase)	[scaled]	Open FAD (Microsolvation)	[scaled]	FAD in Structure 1	[scaled]	Experiment	
C_4_=O	1830 [1757]	C_4_=O, water	1835 [1762]	C_4_=O, water	1804 [1732]	C_4_=O	1712
C_2_=O and H-bridge amine (adenine)	1774 [1703]	C_2_=O, water	1783 [1712]	C_2_=O, water	1758 [1687]	C_2_=O	1657
Adenine	1668 [1600]	Adenine	1700 [1632]	adenine, water clip 1	1680 [1612]	Adenosine	1657
ring I, II, III, amine (adenine)	1638 [1572]	ring I, II, III, amine (adenine)	1640 [1574]	ring I, II, III, water clip 1 & 2, solvating water	1640 [1574]	band I	1626
						Adenosine	1605
ring I, II, III, amine (adenine)	1591 [1527]	ring I, II, III, amine (adenine), solvating water	1594 [1530]	ring I, II, III, water clip 3	1594 [1530]	band II	1580
						band III	1547
						band IV	1506
